# Structural Interface Parameters Are Discriminatory in Recognising Near-Native Poses of Protein-Protein Interactions

**DOI:** 10.1371/journal.pone.0080255

**Published:** 2014-02-03

**Authors:** Sony Malhotra, Kannan Sankar, Ramanathan Sowdhamini

**Affiliations:** National Centre for Biological Sciences (TIFR), GKVK Campus, Bangalore, India; University of South Florida College of Medicine, United States of America

## Abstract

Interactions at the molecular level in the cellular environment play a very crucial role in maintaining the physiological functioning of the cell. These molecular interactions exist at varied levels *viz*. protein-protein interactions, protein-nucleic acid interactions or protein-small molecules interactions. Presently in the field, these interactions and their mechanisms mark intensively studied areas. Molecular interactions can also be studied computationally using the approach named as Molecular Docking. Molecular docking employs search algorithms to predict the possible conformations for interacting partners and then calculates interaction energies. However, docking proposes number of solutions as different docked poses and hence offers a serious challenge to identify the native (or near native) structures from the pool of these docked poses. Here, we propose a rigorous scoring scheme called DockScore which can be used to rank the docked poses and identify the best docked pose out of many as proposed by docking algorithm employed. The scoring identifies the optimal interactions between the two protein partners utilising various features of the putative interface like area, short contacts, conservation, spatial clustering and the presence of positively charged and hydrophobic residues. DockScore was first trained on a set of 30 protein-protein complexes to determine the weights for different parameters. Subsequently, we tested the scoring scheme on 30 different protein-protein complexes and native or near-native structure were assigned the top rank from a pool of docked poses in 26 of the tested cases. We tested the ability of DockScore to discriminate likely dimer interactions that differ substantially within a homologous family and also demonstrate that DOCKSCORE can distinguish correct pose for all 10 recent CAPRI targets.

## Introduction

Protein-protein interactions which constitute the “interactome” of the cell are found in the majority of cellular processes and are known to regulate responses of organisms to the varied environments. Protein molecules in isolation cannot perform any function in the cell; it is the ensemble and their organisation into complexes which maintain the cellular integrity [1]. There are excellent biochemical/experimental techniques, like yeast two hybrid and co-immunoprecipitation, to identify the interacting pairs of proteins. There have been many experimental and computational efforts to use the three-dimensional structures of proteins and identify the interacting pairs of proteins. Molecular docking is a computational technique which is used to predict protein-protein, protein-ligand interactions. It involves the identification of multiple conformations for the interacting partners and then calculating energies for interactions like electrostatics, Van der Waals etc. Initially, the conformational space is searched to find the possible solutions and then these docked poses are scored in order to identify the biological meaningful poses. It is a daunting task to distinguish the biological meaningful poses from a pool of solutions as proposed by the docking algorithm. It would be interesting to computationally predict the interacting pairs of proteins and this will help to gain insights into the molecular interactions behind the cellular process it is involved in.

There are many docking algorithms available like DOCK [2], Autodock [3], ZDOCK [4], FRODOCK [5], GRAMM [6], GOLD [7] etc. As a result of molecular docking, different numbers of docked poses are proposed by the docking algorithms. There is a challenge of scoring these docked poses and hence ranking them to identify the native or near-native structure [8]. In the field, there have been previous attempts to rank the docked poses [9], [10].However, the maximum accuracy achieved is ∼77% and it takes into account only tightness of the interface [9]. Also, conservation of residues has been considered for selecting the near-native conformations from the pool of docked poses [8]. A recent knowledge-based method, DockRank derives the information from homologous interacting proteins for predicting the interface and ranks the docked poses based on the overlap with the predicted interface [11].

When the two components forming the complex interact with each other there is a formation of protein-protein interface. These complexes can be divided into two types based on the nature of interacting partners, homodimers where the two interacting partners are identical and heterodimers where the interacting partners are different. Various properties of this interface formed upon interaction can be utilised to identify the native complex from the pool of proposed docked poses. Here we are describing a new objective scoring scheme named DockScore which takes into account several interface parameters. The weights for these parameters are optimised to improve the accuracy in identifying native or near-native pose.

DockScore aims to determine the optimal interactions by taking into account various parameters of the interface. These parameters include surface area, spatial clustering, conservation of residues, presence of short contacts, hydrophobic residues and positively charged residues at the interface. The scoring method was first trained on a set of 30 non-homologous protein-protein complexes (15 homodimers and 15 heterodimers) to optimise the weights for different parameters. Further, performance of DockScore was assessed on 30 different protein-protein complexes and the native structure (deposited in PDB) or near-native structure was assigned as the best ranked complex from the pool of docked poses.

We further identified some examples of the protein families where different modes of dimerizations are known and tested DockScore for its ability to distinguish these native dimerization modes. It was also tested on 10 of the CAPRI targets.

## Materials and Methods

An objective scoring scheme, DockScore, was developed in order to find the optimal interactions between the two interacting proteins. The scoring scheme was first trained on a set of 30 protein-protein complexes (listed below) and then applied on another set of 30 protein-protein complexes to recognise the best docked pose.

Various features were taken into account and each is described below.

The interface residues were identified using inter-chain C^β^-C^β^ distance cut-off of 7 Å.

### Training and test dataset and generating docked poses

**Figure 1:**
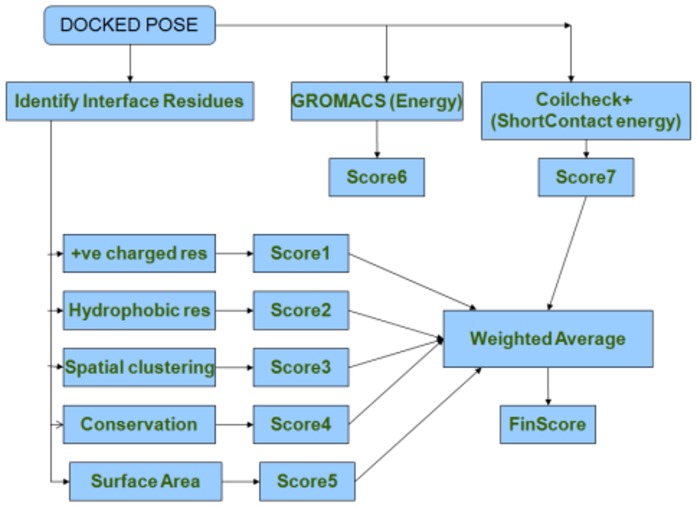
Workflow of DockScore. Interface residues are identified and several structural parameters are taken into account to record the scoring. The final score obtained is the weighted score and weights are assigned by testing the performance. FinScore is the final DockScore obtained using the weighted average.

A dataset of 30 protein-protein complexes was selected from previous studies, which includes 15 homodimers [12] and 15 heterodimers [13]. The protein chains in this dataset do not share more than 25% identity. The two chains from the complexes were segregated into receptor (chain A) and ligand (chain B). The coordinates of receptor chain were altered using the SYBYL software package (Version 7.1) (Tripos Associates Inc.). Further, molecular docking for test dataset was performed using FRODOCK [5] and 99 different docked poses were obtained for all the 30 complexes. The native structure for each of the complex deposited in The Protein Databank (PDB) [14] was added to the pool of 99 docked poses to generate a set of 100 poses for each PDB ID. The overall workflow of DockScore is represented as a schematic in Figure 1.

The same protocol as above was followed to obtain a dataset of 30 protein-protein complexes (15 homodimers and 15 heterodimers) and their docked poses were used to test the performance of DockScore. The chains within and across the two datasets (test and training) do not share more than 25% sequence identity.

### Interface parameters used in scoring

#### Surface Area

Surface area (S.A.) of the interface was calculated using Equation 1.
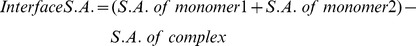
(1)


Surface areas of the monomer and the complex were calculated using NACCESS (Hubbard and Thornton, ‘NACCESS’, Computer Program, 1993) with default parameters and 1.4 Å radius for the probe molecule.

The parameter was scored by assigning maximum score of 1 to the docked pose with highest surface area and minimum score of 0 to the docked pose with minimum surface area (Equation 2).
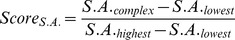
(2)


#### Conservation of residues

Conserved interface residues were identified using ConSurf [15] server. The interface residues (N) from both the chains of the complex with conservation grade of 7, 8 and 9 were considered as conserved interface residues (C_i_).

Scoring based on conservation was performed by assigning high score to the presence of high extent of amino acid conservation at the interface (C_i_) (Equation 3) and normalising it by the number of interface residues (N).
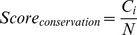
(3)


#### Inter-chain short contacts

Short contacts here imply that two atoms do not closer than the sum of their VanDer Waals' radii. This is to avoid steric clashes and stabilise the complex as a whole. Our program CoilCheck [16] was employed to obtain the Van der Waals interaction energy between the two chains of the protein complex. This energy was used as a measure of the short contacts present at the interface. Since short contacts are undesirable, scores were assigned to the docked pose according to Equation 4, such that pose with high and positive energy obtains low score.
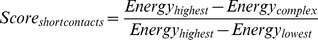
(4)


Here Energy_highest_ is the highest energy value in the pool of 100 poses, Energy_lowest_ is the lowest energy value in the pool of 100 poses and Energy_complex_ is the energy value of the pose being scored.

#### Spatial Clustering

It was employed as a measure of the compactness at the interface. It was calculated for the interface, by computing the pairwise distances between the interface residues between the two chains and the residues with a C^β^-C^β^ distance cut-off of 14 Å were considered as spatially clustered residues. Coefficient of clustering (θ) was calculated using Equation 5, where‘d’ is the number of residues within the cut-off distance and ‘N’ is the number of interface residues at both chains.
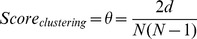
(5)


#### Hydrophobic residues

Protein-protein interfaces of permanently interacting partners are known to be largely hydrophobic in nature [17]–[19]. We ranked those docked poses with high numbers of hydrophobic residues with a high score – as shown in Equation 6, where H_i_ is the number of hydrophobic (A,V, L, I, M, F, W and Y) interface residues and N is the number of interface residues.
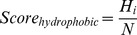
(6)


#### Positively charged residues

In the special case of interaction between DNA-binding proteins or transcription factors, we penalise the presence of positively charged residues at the protein-protein interface using Equation (7), where P_i_ is the number of positively charged residues interface residues (K, H and R) and N is the number of interface residues.
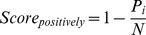
(7)


#### Energy of the docked pose after minimisation

The docked complex was solvated in cubic water box with space water model and box edges 8 Å from molecule periphery. For large complexes, water box with box edges 10 Å from molecule periphery was used. Positive (Na^+^) or negative (Cl^−^) ions were added to achieve charge neutrality. Following this, docked poses were subjected to energy minimisation using software GROMACS 4 [20], employing OPLS force field with 5000 steps of steepest descent. The final energy values were converted to log scale and the scoring was performed using Equation (8) (for explanation of terms, please see equation 4's explanation)
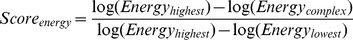
(8)


### Evaluation of the Score

Using all the above mentioned parameters, final score was derived by assigning weights to these parameters. The evaluation was performed in two rounds: in the first round, structures after energy minimisation were subjected to DockScore and in the second round, structures without energy minimisation were used.

The accuracy of the DockScore was measured by enumerating the number of complexes for which the native pose was ranked as the best pose (Equation 9).
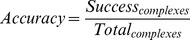
(9)


The weights were assigned to all the seven parameters employing two approaches. Firstly, the ranking of the parameters was done by using Leave-one-out approach and then assigning weights using Rank sum method (Equation 10, where K is the total number of parameters and r_i_ is the rank of i^th^ parameter).
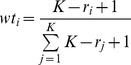
(10)


Secondly, each parameter was assessed by using only that parameter alone and calculating the number of correct predictions. The weights derived were further normalised by the total sum of weights.

### Proteins with different dimerization modes

The performance of DockScore was further tested on proteins belonging to same family and possessing different dimerization modes: GAF proteins, IL-8 superfamily proteins, DJ-1 superfamily and H-NS histone like proteins superfamily. For the different modes of dimerization known, the structures were obtained from PDB and a similar methodology was adopted to perform docking and then the docked poses were subjected to DockScore to access if it was able to distinguish between the different dimerization modes.

### Testing on earlier CAPRI targets

Ten of the CAPRI targets were selected to test the preformance of DockScore. These were selected to include enzyme-inhibitor (3), antigen-antibody (2) and other protein-protein complexes (5).

## Results

### Evaluation of DockScore performance on training set

DockScore was first trained on a set of 30 protein-protein complexes (Tables 1 and 2) and weights were assigned to different parameters used in scoring. 99 docked poses were generated for each of the 30 complexes using FRODOCK docking tool.

**Table 1:** List of 15 homodimers (chain A and B) used for training the scoring scheme.

PDB ID	Name of the complex
1A4I	Human tetrahydrofolate dehydrogenase/cyclohydrolase
1B3A	Total chemical synthesis and high-resolution crystal structure of the potent anti-hiv protein aop-rantes
1BD0	Alanine racemase complexed with alanine phosphonate
1BKP	Thermostable thymidylate synthase A from *Bacillus subtilis*
1BXK	Dtdp-glucose 4,6-dehydratase from *E. coli*
1CHM	Enzymatic mechanism of creatine amidinohydrolase as deduced from crystal structures
1FIP	The structure of fis mutant pro61ala illustrates that the kink within the long alpha-helix is not due to the presence of the proline residue
1HXP	Nucleotide transferase
1IVY	Physiological dimer hpp precursor
1OAC	Crystal structure of a quinoenzyme: copper amine oxidase of *Escherichia coli*
1R2F	Ribonucleotide reductase r2f protein from *Salmonella typhimurium*
1SMN	Identification of the serratia endonuclease dimer: structural basis and implications for catalysis
1VFR	The major NAD(P)H:FMN oxidoreductase from V*ibrio fischeri*
2SPC	Crystal structure of the repetitive segments of spectrin
5RUB	Crystallographic refinement and structure of ribulose-1,5-bisphosphate carboxylase from *Rhodospirillum rubrum*

**Table 2:** List of 15 heterodimers (chain A and B) used for training DockScore.

PDB ID	Name of the complex
1ABR	Crystal structure of Abrin-a
1BND	Structure of the brain-derived neurotrophic factor/neurotrophin 3 heterodimer
1DHK	Structure of porcine pancreatic alpha-amylase
1E96	Structure of the rac/p67phox complex
1F2T	Crystal Structure of ATP-Free RAD50 ABC-ATPase
1FT1	Crystal structure of protein farnesyltransferase
1JKG	Structural basis for the recognition of a nucleoporin FG-repeat by the NTF2-like domain of TAP-p15 mrna nuclear export factor
1KSH	Complex of Arl2 and PDE delta, Crystal Form 2 (native)
1MA9	Crystal structure of the complex of human vitamin D binding protein and rabbit muscle actin
1OHZ	Cohesin-dockerin complex from the cellulosome of *Clostridium thermocellum*
1PQZ	Murine cytomegalovirus immunomodulatory protein m144
1SLU	Rat anionic n143h, e151h trypsin complexed to a86h ecotin
1US7	Complex of hsp90 and p50
1TX4	Rho/rhogap/gdp(dot)alf4 complex
1L4Z	X-ray crystal structure of the complex of microplasminogen with alpha domain of streptokinase in the presence cadmium ions

DockScore takes into account several parameters of the interface. Firstly, it identifies the interface residues and then scores for conservation, short contacts, spatial clustering and the nature of residues at the interface (see Materials and Methods).

When the energy minimised poses (obtained using GROMACS v 4.5) were used, and the weights were assigned using one parameter at a time (Table S1). However, the accuracy observed was only ∼27%.

Subsequently, the poses were examined without being subjected to energy minimisation and the performance of scoring was assessed by enumerating the complexes where DockScore accurately assigned to rank to the native form. Assigning equal weights to all the parameters resulted in an accuracy of 77% (p-value = 0.0002). Next, the scores were assigned by removing one parameter at a time (Table S2) and also by using only one parameter at a time (Table 3). The weights, when assigned employing the latter approach, resulted in an accuracy of 96% (29 out of 30 protein-protein complexes could be identified well).

**Table 3:** Weights assigned to different parameters using one parameter at a time.

Parameter used (only)	Correct Prediction Homodimers	Correct Prediction Heterodimers	Total correct predictions	Weights assigned	Normalized weights
A	2	6	8	0.27	0.20
B	14	15	29	0.97	0.73
C	1	1	2	0.07	0.05
D	0	0	0	0	0
E	0	1	1	0.03	0.02
F	0	0	0	0	0

Weights were assigned to different parameters A. Interface Surface area, B. Short contacts at interface, C. Conservation at interface, D. Spatial Clustering at the interface, E. Interface Hydrophobicity and F. Positively charged residues at the interface, by using only one parameter at a time and assessing the importance of each parameter by counting the total number of correct predictions were observed.

### Performance of DockScore on test dataset

After training the scoring scheme on a set of 30 protein-protein complexes, we selected another set of 30 complexes (Table 4 and 5) to test its performance and to assess the weights for each parameter which were optimised using training dataset. The 30 protein-protein complexes in the test dataset were kept different from those in the training dataset (please see Methods; Figure S1 and S2 reflect this in form of principal component analysis : PCAplot).

**Table 4:** List of 15 homodimers (chain A and B) used for testing DockScore.

PDB ID	Name of the complex
12AS	Asparagine synthetase mutant c51a, c315a complexed with l-asparagine and amp
1AA7	Influenza virus matrix protein crystal structure at ph 4.0
1BO4	Crystal structure of a gcn5-related n-acetyltransferase: *Serratia marescens* aminoglycoside 3-n acetyltransferase
1CI4	The crystal structure of human barrier-to-autointegration factor (baf)
1CQ3	Structure of a soluble secreted chemokine inhibitor, vcci, from cowpox virus
1CRU	Soluble quinoprotein glucose dehydrogenase from *Acinetobacter calcoaceticus* in complex with pqq and methylhydrazine
1DK0	Crystal structure of the hemophore hasa from Serratia marcescens crystal form p2(1), ph 8
1G58	Crystal structure of 3,4-dihydroxy-2-butanone 4-phosphate synthase gold derivative
1QMH	Crystal structure of RNA 3′-terminal phosphate cyclase, an ubiquitous enzyme with unusual topology
2AIB	Beta-cinnamomin in complex with ergosterol
2ARC	*Escherichia coli* regulatory protein AraC complexed with L-arabinose
2LIG	Three-dimensional structures of the ligand-binding domain of the bacterial aspartate receptor with and without a ligand
2Q3A	Crystal structure of Rhesus macaque CD8 alpha-alpha homodimer
2QFR	Crystal structure of red kidney bean purple acid phosphatase with bound sulfate
4FTX	Crystal structure of ego3 homodimer

**Table 5:** List of 15 heterodimers (chain A and B) used for testing DockScore.

PDB ID	Name of the complex
1BH8	HTAFII18/HTAFII28 heterodimer crystal structure
1DF0	Crystal structure of M-Calpain
1EUV	X-ray structure of the C-terminal ULP1 protease domain in complex with SMT3, the yeast ortholog of SUMO
1GHD	Crystal structure of the glutaryl-7-aminocephalosporanic acid acylase by mad phasing
1JEQ	Crystal Structure of the Ku Heterodimer
1L5H	FeMo-cofactor deficient nitrogenase MoFe Protein
1SB2	High resolution structure determination of rhodocetin
2AHO	Structure of the archaeal initiation factor eIF2 alpha-gamma heterodimer from *Sulfolobus solfataricus* complexed with GDPNP
2C0J	Crystal tructure of the BET3-TRS33 heterodimer
2HLE	Structural and biophysical characterization of the EPHB4-EPHRINB2 protein protein interaction and receptor specificity
2OT3	Crystal structure of rabex-5 VPS9 domain in complex with nucleotide free RAB21
2OVP	Structure of the Skp1-Fbw7 complex
2VLQ	F86 mutant of E9 DNAse domain in complex with IM9
3H7W	Crystal structure of the high affinity heterodimer of HIF2 alpha and ARNT C-terminal PAS domains with the artificial ligand THS017
3SDE	Crystal structure of a paraspeckle-protein heterodimer, PSPC1/NONO

**Figure 2:**
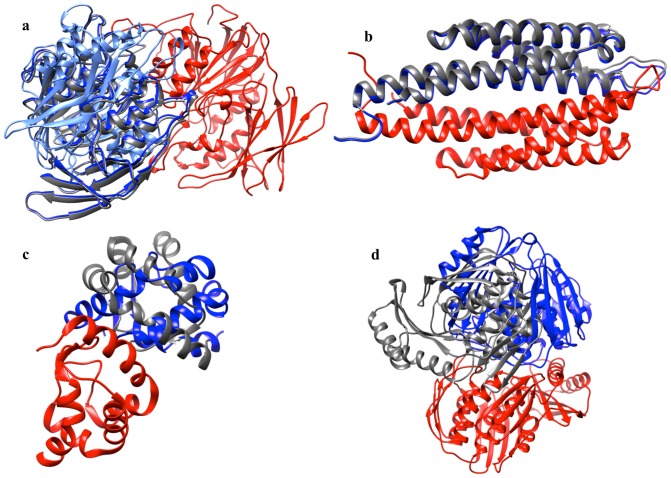
DockScore performance on testing dataset. 15 heterodimers and 15 homodimers were used for testing the performance of the scoring scheme. DockScore assigned the top-most rank in 26 of the cases. The four cases where, non-native pose was assigned a higher score than the native pose were analysed by structural superposition, (a) 2QFR, (b) 2LIG, (c) 2AIB and (d) 1QMH. One of the chain of all poses, including the native, is colored red, native pose of the other chain in grey and the non-native poses which were ranked higher are shown in blue colour. These poses were observed to be structurally very similar to native pose.

DockScore was able to distinguish the native pose from a pool of docked poses in 26 of the complexes in the test dataset (87% accuracy, these 4 cases belong to homodimers set). In the cases, where non-native pose was ranked higher than the native, there was a small difference (0.02–0.1) in the scores as assigned by DockScore. The non-native top scoring poses were observed to be structurally similar to the native pose (Figure 2). We further, calculated the fraction of overlap of the interface residues between the native and the top-ranked non-native pose. We observed that this fraction of overlap lies in the range of 0.32–0.98.

**Figure 3:**
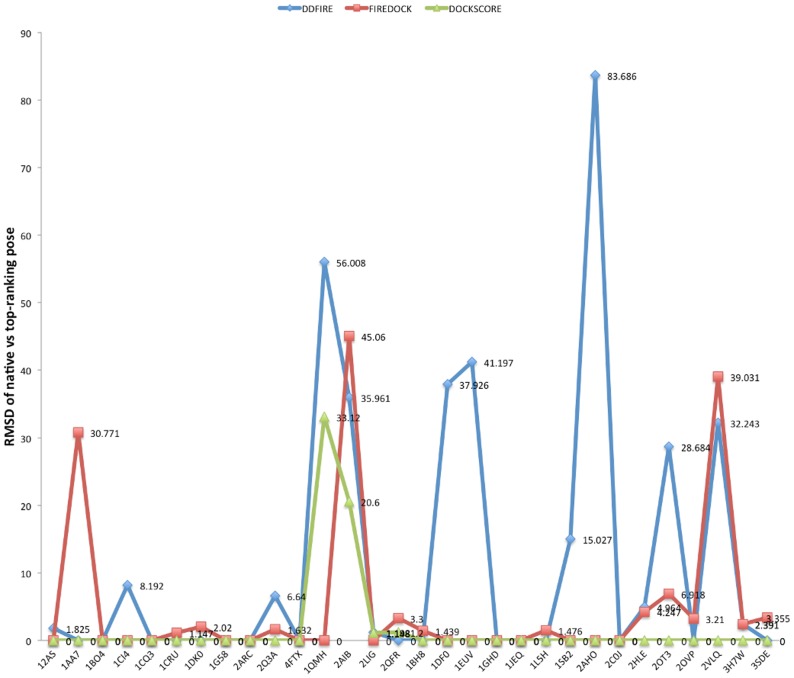
Comparison of DockScore performance with other methods. The ability of DockScore to rank native structure as the top-most pose, was compared with two of the other methods namely dDFIRE and FireDock. The native structure was assigned top-most rank in 26, 16 and 16 complexes with DockScore, dDFIRE and FireDock respectively. The all-atom RMSD for smaller chain was computed between the native structure and the top-ranking pose as identified using the three methods.

We further compared the performance of DockScore with two of the previously reposrted scoring methods namely dDFIRE [21] and FireDock [22]. As mentioned before, DockScore ranked native structure as top-most structure for 26 of the complexes (for rest four cases, refer Figure 2: top-ranking pose is structurally similar to the native complex structure), whereas dDFIRE and FireDock report this in 16 of the complexes (Figure 3).

### Superfamilies with different dimerization modes

DockScore was further assessed for its performance on the four unrelated superfamilies whose members are known to possess different dimerization modes: GAF proteins, IL-8 superfamily proteins, DJ-1 superfamily and H-NS histone-like proteins superfamily.

GAF domains are present in cGMP regulated cycilc nucleotides phosphodiesterases, certain adenylyl cyclases and bacterial TF FhlA (formate hydrogen lyase transcription activator) [23]. We selected three structures of proteins possessing GAF domains and having different modes of dimerization (1YKD, 1MC0 and 1F5M). 1YKD is a 1.9 Å resolution crystal structure of cyaB2/GAF A and GAF B antiparallel dimer from *Anaebaena* [24], 1MC0 is 2.9 Å PDE2/GAF A and GAF B parallel dimer from *Mus musculus* [25] and 1F5M is 1.9 Å crystal structure of YKG9 protein from *S.cerevisiae* [23].

DJ-1 superfamily of proteins is known to exhibit different dimerization modes [26], there are four distinct patches of interface known in this superfamily. We selected a protein dimer complex corresponding to each of the four distinct types of dimerization modes to exemplify four respective interface patches (1IZY, 2VRN, 1QVV and 1VHQ).

SCOP also documents different modes of dimerization in certain superfamilies, like H-NS histone like proteins superfamily and IL-8 superfamily. We selected proteins with different modes of dimerization known in these superfamilies (for IL-8 like superfamily: 2IL8, 1DOK and for H-NS superfamily: 1OV9, 1LR1).

Using one chain as receptor and another as ligand, 99 docked poses were generated for each of the different complexes, employing FRODOCK. These 99 docked poses and the native structure, were subjected to DockScore.

For two of the superfamilies, IL-8 like and H-NS histone like proteins superfamily, DockScore accurately scores the native pose as the top ranked pose. In the case of GAF domains, out of the three different dimerization modes tested, DockScore accurately scores native pose as top-ranked pose in two modes. In the DJ-1 superfamily, out of the four different modes tested, native poses in two modes were accurately ranked as the top pose using DockScore. These cases, where non-native pose was ranked higher than the native, were further analysed.

**Figure 4:**
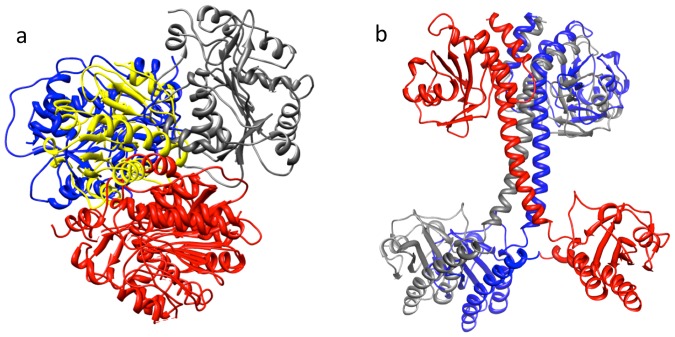
DockScore performance on the superfamilies known to possess different modes of dimerization. DockScore was tested for its ability to distinguish the different modes of dimerization in four of the superfamilies, namely GAF domains, IL-8 like superfamily, DJ1 superfmily and H-NS histone like proteins. For the superfamily DJ-1, out of the four different known dimerization modes, DockScore assigned top rank to native pose in two of the modes. One of the chains is shown in red, the native pose of the other chain in grey and the non-native pose in blue colour. (a) In one of the modes (1IZY), we observed that the non-native pose, obtaining the highest score, was structurally very similar to the native pose observed in another dimerization mode of the same superfamily (1VHQ). (b) In GAF domains, 1MC0, the top-ranked pose was very similar to the native pose. In yellow is another type of dimerization mode observed in DJ-1 superfamily.

For the DJ-1 superfamily, the top-ranked pose and the native pose were very similar and they differ in score by only 0.1 (Figure 4a) and the observed top-ranked pose in one of the dimerization mode (1IZY) also closely resembles the native dimerization mode seen in 1VHQ (Figure 4a). In one of the high-scoring trials corresponding to the dimerization mode of GAF domains, 1MC0, the difference in the scores of the native pose and the top-ranked pose was 0.2 but the interface of the top-ranked pose and the native pose were observed to share significant overlap (Figure 4b).

### Proteins in bound and unbound forms

In order to examine the effect of conformational changes and large-scale rotations that might accompany during interactions with other proteins, we next chose five proteins that are available in the bound as well as protein-unbound form as compiled in ComSin database [27]. DOCKSCORE could successfully rank the crystal structure as the highest and identify the best docked pose with high rank for the five proteins when started from the bound-conformation of the protein (Table S3). However, this happened less easily and only for two cases, when starting from the unbound-conformation of the protein confirming that recognition of the correct docking pose can remain a bottle-neck where there are substantial structural changes during protein-protein interactions.

### Testing on CAPRI targets

DockScore performance was further evaluated on 10 of the earlier CAPRI targets (Table 6). This was performed to understand the sensitivity of DOCKSCORE in scoring different docked poses in CAPRI targets. Different docked poses were obtained using FRODOCK (as explained earlier for the training and testing datasets) and the native pose was included as in PDB file.

**Table 6:** List of 10 earlier CAPRI targets (with chain identifiers in parentheses) employed to evaluate DockScore performance.

PDB ID	PDB name	Score of the native pose	Difference in Native and top ranking pose
1KEN (A–B)	Influenza virus hemagglutinin complexed with and antibody that prevents the hemagglutinin low pH fusogenic transition	0.92	-
1KXT (A–B)	Camelid VHH Domains in Complex with Porcine Pancreatic alpha-Amylase	0.95	-
1SYX (A–B)	The crystal structure of a binary U5 snRNP complex	0.89	-
1TA3 (A–B)	Crystal Structure of xylanase (GH10) in complex with inhibitor (XIP)	0.77	0.07
1TE1 (A–B)	Crystal structure of family 11 xylanase in complex with inhibitor (XIP-I)	0.88	-
1URZ (A–B)	Low pH induced, membrane fusion conformation of the envelope protein of tick-borne encephalitis virus	0.94	-
1V74 (A–B)	Ribonuclease-inhibitor complex	0.93	-
2VDU (B–E)	Structure of TRM8-TRM82, the yeast tRNA M7G methylation complex	0.83	-
3R2X (A–B)	Crystal structure of the de novo designed binding protein HB36.3 in complex with the 1918 influenza virus hemagglutinin	0.87	0.05
3U43 (A–B)	Crystal structure of the colicin E2 DNase-Im2 complex	0.88	-

**Figure 5:**
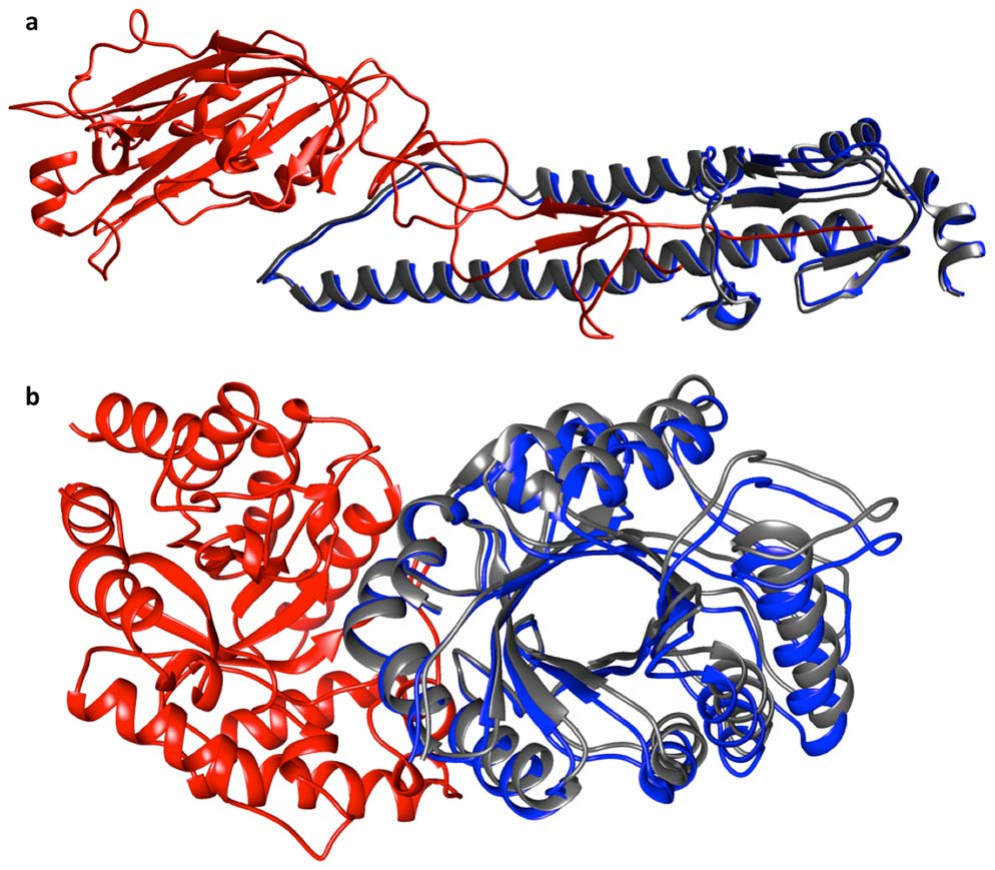
Evaluation of the performance of DockScore on CAPRI targets. DockScore was also tested on 10 of the earlier CAPRI targets. In two of the cases, the top-ranked pose was structurally very similar to the native pose. (a) 3R2X and (b) 1TA3. One of the chains of all poses, including the native, is colored red, native pose of the other chain in grey and the top-ranking non-native pose of the other chain in blue.

We were able to assign the top-most rank to the native structure in 8 out of 10 targets. In remaining two cases (3R2X and 1TA3), where some docked pose obtained a higher score than native, were further analysed for the structural proximity of high-ranking docked pose to the native pose. These poses were observed to be well-superposed with the native pose (Figure 5) and the difference in the scores of the native and the top-ranking non-native poses were 0.05 and 0.07 respectively. Therefore, we were able to accurately distinguish the native (or near-native) pose, out of a pool of docked complexes in all 10 of the CAPRI targets (Table S4).

## Discussion

Since accurate structure determination of the macromolecular complexes is highly challenging and biologically important, prediction of protein-protein interactions through molecular docking is highly appropriate. However, the implementation of molecular docking for studying the interactions between a pair of proteins poses a challenge to identify the best docked pose out of the pool of various poses suggested by the docking program. For identifying the best docked pose, we devised an objective scoring scheme, named DockScore, which takes into account several interface parameters and hence ranks the docked poses.

The interface residues are first identified for each pose and the docked poses are ranked based on the parameters like interface surface area, spatial clustering at the interface, presence of hydrophobic, positively charged residues, conservation of residues and undesirable short contacts at the interface. The contribution of the energy of each pose was also tested by subjecting the energy minimised poses for scoring to DockScore. Before testing the performance of the scoring scheme for selecting the best pose, it was first trained on 30 protein-protein complexes. The weights were assigned, using one parameter at a time, indicating the importance of each parameter in the final score and thereby accuracy could be tuned close to 96%. Subsequently, it was tested for its accuracy on a test dataset, which comprised of 30 other complexes.

There were several tests adopted to assess the performance of DockScore:

1. The scoring was considered accurate, when DockScore was able to rank the native structure (deposited in PDB) or near-native structure as the best rank pose.
2. If the non-native poses were ranked higher than the native pose, we further analysed the difference in their scores assigned by DockScore and the fraction of overlapping interface residues.
3. DockScore was further tested on four superfamilies, where different dimerization modes are known, for its ability to distinguish the native pose from a pool of docked poses.
4. This scoring scheme when further applied on 10 of the earlier CAPRI targets was able to distinguish the native pose from the pool of 100 docked poses in all 10 targets.

In the present study, FRODOCK has been used to obtain close to 100 docked poses. However, the objective scoring scheme devised can be used in general to rank the docked poses suggested by any docking program and for a variety of protein-protein interactions. In the near future, DockScore would be tested on other protein-protein complexes and other docking programs can also be used to test the accuracy of DockScore.

These kinds of studies will aim to provide detailed insights into the interactions amongst proteins which are quite direct in nature. Also, the existence of interactions among protein factors regulating the direct interactions will provide an additional level of regulation on one hand, whereas it will also lead to the combinatorial diversity of regulatory complexes. With different combinations of these factors, regulation of diverse numbers of genes can be achieved. Therefore, studying the physical interactions amongst proteins will provide useful insights into unravelling the basis of this combinatorial diversity in eukaryotes.

## Supporting Information

Figure S1 **Principal component analysis of 15 homodimers in the training set (corresponding to** **Table 1** **) and 15 homodimers in the test set (corresponding to** **Table 4** **).** High dispersion shows that there is no bias or high sequence identity across the training and test dataset.
(TIF)

Figure S2 **Principal component analysis of 15 heterodimers in the training set (corresponding to** **Table 2** **) and 15 heterodimers in the test set (corresponding to** **Table 5** **).** High dispersion shows that there is no bias or high sequence identity across the training and test dataset.
(TIF)

Table S1 **Weights assigned to different parameters using energy minimized structures.**
(DOC)

Table S2 **Weights assigned to different parameters removing one parameter at a time.**
(DOC)

Table S3 **Five cases (both bound and unbound form) from ComSin database, which were subjected to DockScore.** All the five cases are validated using PISA as well. (*starting from unbound monomeric forms).
(DOCX)

Table S4 **Results of the application of DockScore on 10 CAPRI targets.**
(XLS)
