# Supplementary figures and images for: Structural Interface Parameters Are Discriminatory in Recognising Near-Native Poses of Protein-Protein Interactions
Source: PLoS One. 2014 Feb 3;9(2):e80255. doi: 10.1371/journal.pone.0080255 (PMC3912216; doi:10.1371/journal.pone.0080255)

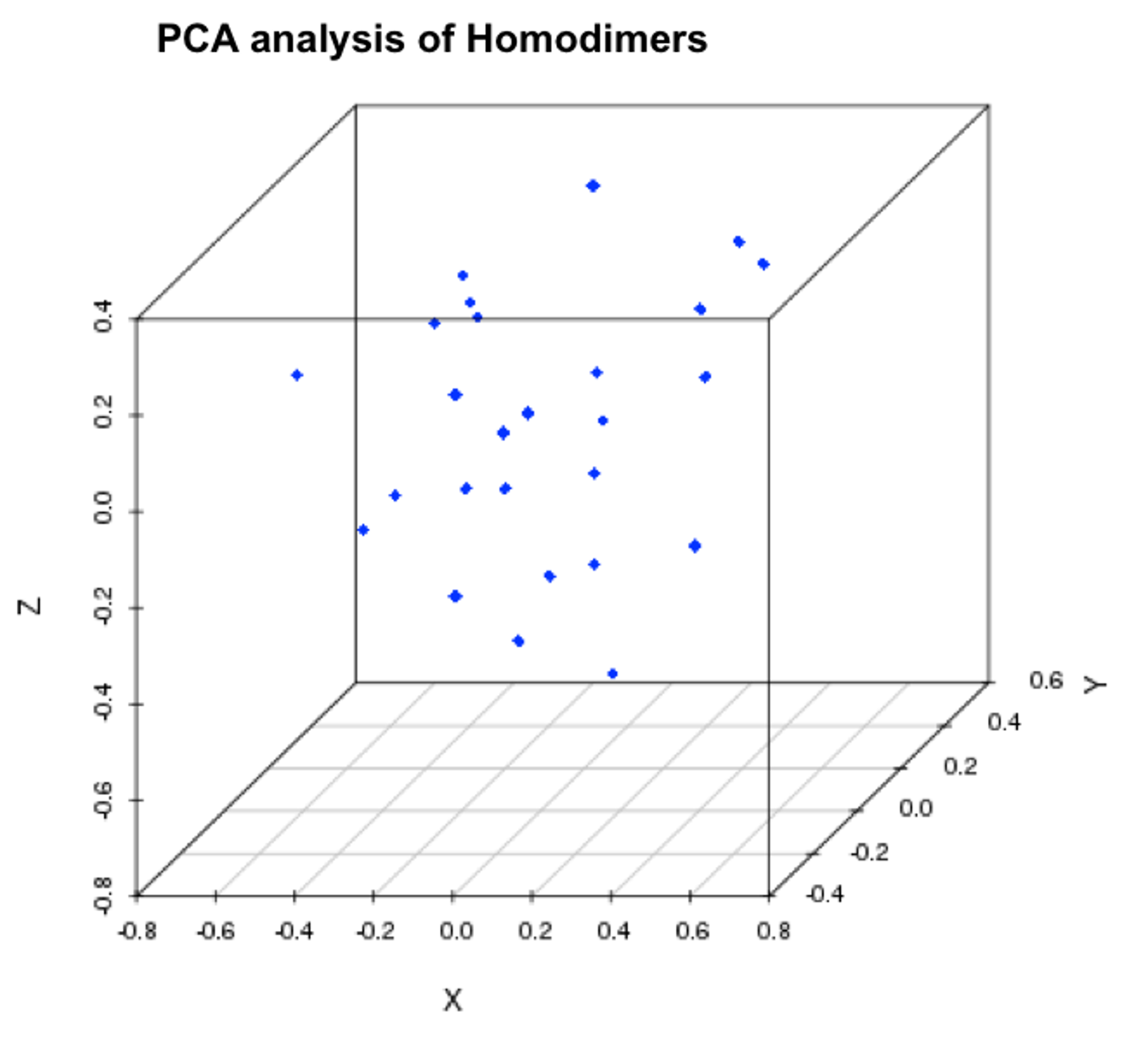

Supplement: Figure S1 — Principal component analysis of 15 homodimers in the training set (corresponding to Table 1 ) and 15 homodimers in the test set (corresponding to Table 4 ). High dispersion shows that there is no bias or high sequence identity across the training and test dataset. (TIF) [file pone.0080255.s001.tif]

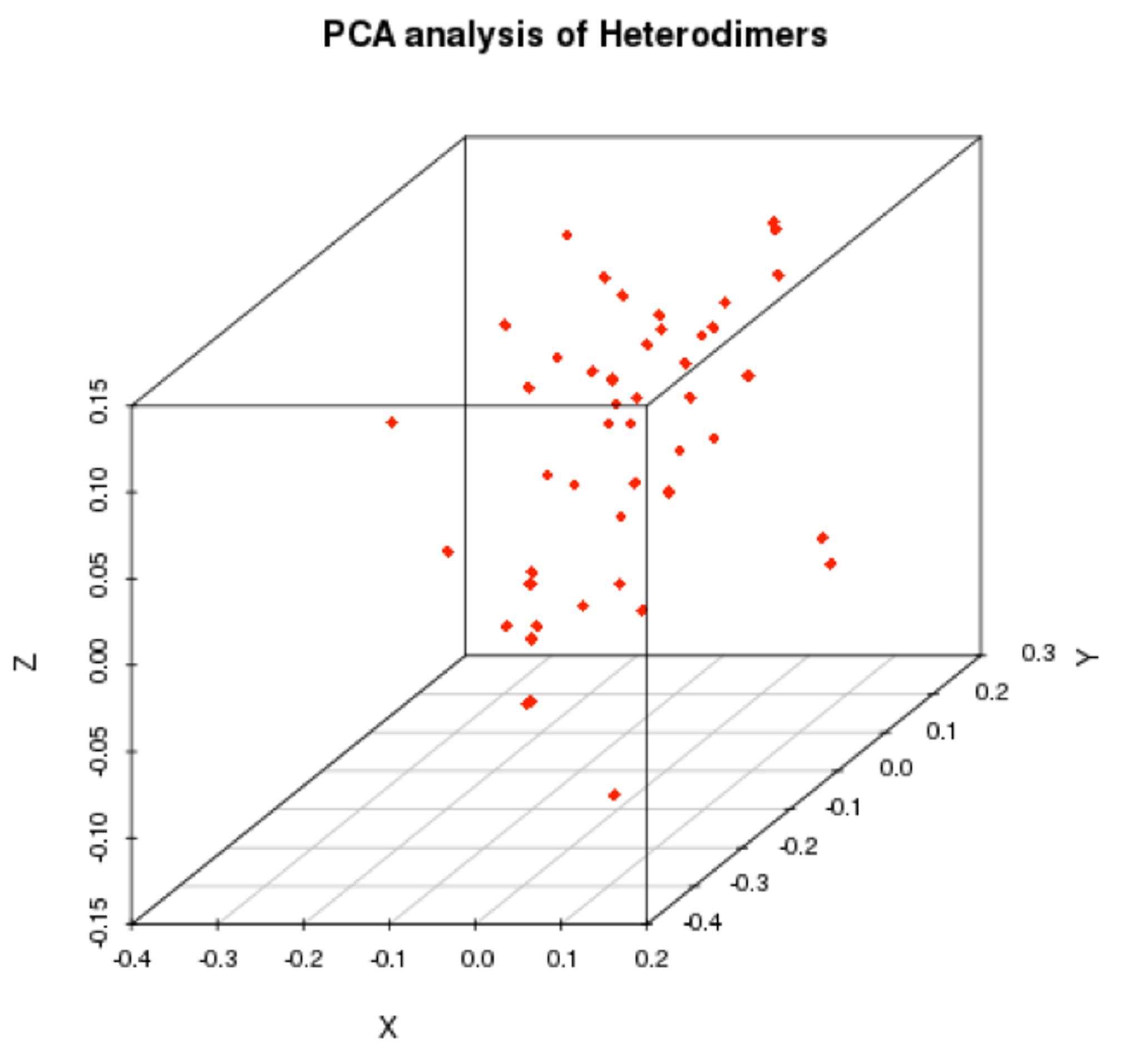

Supplement: Figure S2 — Principal component analysis of 15 heterodimers in the training set (corresponding to Table 2 ) and 15 heterodimers in the test set (corresponding to Table 5 ). High dispersion shows that there is no bias or high sequence identity across the training and test dataset. (TIF) [file pone.0080255.s002.tif]
