# Supplementary material for: Structural Interface Parameters Are Discriminatory in Recognising Near-Native Poses of Protein-Protein Interactions
Source: PLoS One. 2014 Feb 3;9(2):e80255. doi: 10.1371/journal.pone.0080255 (PMC3912216; doi:10.1371/journal.pone.0080255)
Supplement: Table S1 — Weights assigned to different parameters using energy minimized structures. (DOC) [file pone.0080255.s003.doc]

Weights were assigned to seven different parameters A. Interface Surface area, B. Short contacts at interface, C. Conservation at interface, D. Spatial Clustering at the interface, E. Interface Hydrophobicity, F. Positively charged residues at the interface and G. Energy of the complex, by using only one parameter at a time and assessing the importance of each parameter by counting the total number of successes observed.

**Table S1.**

Weights assigned to different parameters using energy minimized structures.

| Parameter used (only) | Successes  Homodimers | Successes  Heterodimers | Total successes | Weights assigned | Normalized weights |
| --- | --- | --- | --- | --- | --- |
| Interface Surface area | 6 | 2 | 8 | 0.264 | 0.35 |
| Short contacts at interface | 8 | 3 | 11 | 0.363 | 0.48 |
| Conservation at interface | 1 | 0 | 1 | 0.033 | 0.04 |
| Spatial Clustering at the interface | 0 | 0 | 0 | 0 | 0 |
| Interface Hydrophobicity | 1 | 0 | 1 | 0.033 | 0.04 |
| Positively charged residues at the interface | 0 | 1 | 1 | 0.033 | 0.04 |
| Energy of the complex | 1 | 0 |  | 0.033 |  |
