# Supplementary material for: Structural Interface Parameters Are Discriminatory in Recognising Near-Native Poses of Protein-Protein Interactions
Source: PLoS One. 2014 Feb 3;9(2):e80255. doi: 10.1371/journal.pone.0080255 (PMC3912216; doi:10.1371/journal.pone.0080255)
Supplement: Table S2 — Weights assigned to different parameters removing one parameter at a time. (DOC) [file pone.0080255.s004.doc]

Weights were assigned to different parameters A. Interface Surface area, B. Short contacts at interface, C. Conservation at interface, D. Spatial Clustering at the interface, E. Interface Hydrophobicity and F. Positively charged residues at the interface, by removing one parameter at a time, assessing the importance of each parameter by counting the total number of successes observed and using Rank sum method (see text).

**Table S2.**

Weights assigned to different parameters removing one parameter at a time.

| Parameter removed | Total successes | Rank of parameter | Weights assigned |
| --- | --- | --- | --- |
| Interface Surface area | 8 | 1 | 0.261 |
| Short contacts at interface | 9 | 2 | 0.217 |
| Conservation at interface | 21 | 3 | 0.174 |
| Spatial Clustering at the interface | 21 | 3 | 0.130 |
| Interface Hydrophobicity | 23 | 4 | 0.130 |
| Positively charged residues at the interface | 24 | 5 | 0.087 |
