# Supplementary material for: Structural Interface Parameters Are Discriminatory in Recognising Near-Native Poses of Protein-Protein Interactions
Source: PLoS One. 2014 Feb 3;9(2):e80255. doi: 10.1371/journal.pone.0080255 (PMC3912216; doi:10.1371/journal.pone.0080255)
Supplement: Table S3 — Five cases (both bound and unbound form) from ComSin database, which were subjected to DockScore. All the five cases are validated using PISA as well. (*starting from unbound monomeric forms). (DOCX) [file pone.0080255.s005.docx]

| Bound form | Unbound form | RMSD between bound and unbound forms | % Interface overlap between top-ranking and native pose* |
| --- | --- | --- | --- |
| 1rrg (A, B) | 1rrf (A) | 0.85 | - |
| 2g1j (A, B) | 1l4u (A) | 1.08 | - |
| 1cbi (A, B) | 2cbr (A) | 1.57 | 47.3 |
| 2nrf (A, B) | 2ic8 (A) | 1.94 | 38.2 |
| 1lyz (A, B) | 1lis (A) | 0.69 | - |

Table S3: Five cases (both bound and unbound form) from ComSin database, which were subjected to DockScore. All the five cases are validated using PISA as well. (*starting from unbound monomeric forms)
